# Semantic Segmentation Network Based on Adaptive Attention and Deep Fusion Utilizing a Multi-Scale Dilated Convolutional Pyramid

**DOI:** 10.3390/s24165305

**Published:** 2024-08-16

**Authors:** Shan Zhao, Zihao Wang, Zhanqiang Huo, Fukai Zhang

**Affiliations:** School of Software, Henan Polytechnic University, Jiaozuo 454000, China

**Keywords:** semantic segmentation, adaptive attention, feature fusion, global average pooling

## Abstract

Deep learning has recently made significant progress in semantic segmentation. However, the current methods face critical challenges. The segmentation process often lacks sufficient contextual information and attention mechanisms, low-level features lack semantic richness, and high-level features suffer from poor resolution. These limitations reduce the model’s ability to accurately understand and process scene details, particularly in complex scenarios, leading to segmentation outputs that may have inaccuracies in boundary delineation, misclassification of regions, and poor handling of small or overlapping objects. To address these challenges, this paper proposes a Semantic Segmentation Network Based on Adaptive Attention and Deep Fusion with the Multi-Scale Dilated Convolutional Pyramid (SDAMNet). Specifically, the Dilated Convolutional Atrous Spatial Pyramid Pooling (DCASPP) module is developed to enhance contextual information in semantic segmentation. Additionally, a Semantic Channel Space Details Module (SCSDM) is devised to improve the extraction of significant features through multi-scale feature fusion and adaptive feature selection, enhancing the model’s perceptual capability for key regions and optimizing semantic understanding and segmentation performance. Furthermore, a Semantic Features Fusion Module (SFFM) is constructed to address the semantic deficiency in low-level features and the low resolution in high-level features. The effectiveness of SDAMNet is demonstrated on two datasets, revealing significant improvements in Mean Intersection over Union (MIOU) by 2.89% and 2.13%, respectively, compared to the Deeplabv3+ network.

## 1. Introduction

Image semantic segmentation assigns a semantic category to each input image pixel to obtain pixel-wise dense classification. This is a fundamental task in computer vision and is vital in various applications such as medical image analysis [1], target detection [2], and autonomous driving [3]. Semantic segmentation methods can be categorized into traditional and deep neural network-based methods.

The former methods primarily rely on image features such as texture, color, shape, and edges, dividing the image into regions with similar characteristics through different algorithms. Traditional image segmentation methods involve techniques based on thresholds [4], edges [5], and regions [6]. Threshold-based image segmentation methods typically classify image pixels into foreground and background by setting a grayscale threshold and categorizing the image’s grayscale histogram. Although this strategy is simple to implement and computationally efficient, the segmentation results on complex images are poor. Region-based segmentation methods segment images based on spatial information, classify pixels according to pixel similarity features, and form regions. Although such a segmentation process suits complex images, region-based segmentation methods are sensitive to noise, under-segmenting or over-segmenting images. Moreover, edge-based image segmentation methods determine potential boundaries by detecting differences in grayscale values between pixels and adjacent pixels and connecting these boundaries to form edge contours. Typical edge detection operators are Roberts [7], Sobel [8], Prewitt [9], LoG [10], and Canny [11], which, although they produce relatively clear edge contours, they are sensitive to noise and do not consider regional information between pixels, leading to over-segmentation. Despite traditional semantic segmentation methods performing exceptionally well on images with a uniform grayscale or slight differences in the grayscale between the object and background, they overlook the image’s semantic, spatial, and other feature information. Furthermore, these methods are susceptible to noise and thus unsuitable for complex images. In conclusion, traditional approaches rely heavily on handcrafted features and suffer from the abovementioned drawbacks. To provide a clearer understanding of these traditional methods, a summary table is presented below (Table 1).

Neural network technology has recently become mainstream in image segmentation, aiming to overcome these limitations. For instance, AlexNet [12] employs multiple convolutions to extract local features, significantly improving segmentation beyond traditional methods. Regarding semantic segmentation, dual-branch structured networks have been widely utilized. For instance, BiSeNet [13] introduces a detail branch that focuses on extracting details and boundary information and complements the semantic branch, which captures global contextual information. This dual-branch network enriches the spatial features. Additionally, BiSeNetv2 [14] introduces a powerful detail branch and lightweight spatial branch on top of BiSeNet, realizing better performance and real-time inference speed. Similarly, DANet [15] integrates local and global features using spatial and channel attention modules, enhancing feature representation for precise segmentation without relying on multi-scale feature fusion. Furthermore, ACNet [16] utilizes Asymmetric Convolution Blocks (ACBs) to replace standard convolutions, enhancing accuracy and robustness to distortions and seamlessly integrating into existing architectures for improved performance. Moreover, OCRNet [17] aggregates object-contextual representations to enhance pixel classification in semantic segmentation by leveraging the relationship between pixels and object regions, resulting in improved segmentation accuracy. In addition, BiMSANet [18] tackles scale variation in oblique aerial images by using bidirectional multi-scale attention networks, which fuse features adaptively for more effective semantic segmentation. Twins [19] revisits spatial attention design in vision transformers, proposing efficient architectures Twins-PCPVT and Twins-SVT, excelling in classification, detection, and segmentation with optimized matrix multiplications. HMANet [20] improves VHR aerial image segmentation by integrating class-augmented attention and region shuffle attention to effectively capture global correlations, enhancing efficiency and performance on benchmark datasets. Meanwhile, DDRNet [21] effectively fuses the features through bidirectional and dense connections and introduces a multi-scale feature fusion technique that enhances the network’s ability to process multi-scale features. Neural network models aim to improve segmentation accuracy through multi-scale feature fusion and information recovery.

Encoder–decoder structures have also been widely used in image segmentation. For example, U-Net [22] is known for its encoder–decoder architecture. It extracts features through the encoder, restores the original resolution through the decoder, and ingeniously utilizes skip connections to fuse low-level and deep-level information. Moreover, U2-Net [23] adopts a two-level nested U-shaped structure, with each encoder and decoder module resembling UNet. Residual U blocks (RSU) with different-sized receptive fields are adopted to capture more contextual information while pooling operations are also employed to increase the network’s depth. This architecture effectively improves its performance, reduces training costs, and solves the problems of insufficient scale and contextual information in image segmentation. Similarly, SegFormer [24] advances the field by unifying Transformers with MLP decoders, using a hierarchically structured encoder and lightweight decoder to efficiently aggregate multi-scale features. This approach achieves superior performance without relying on positional encoding, marking a significant step forward. In addition, UVid-Net [25] enhances UAV video semantic segmentation by incorporating temporal information into an encoder–decoder CNN. Additionally, a feature-refiner module is added to ensure accurate, temporally consistent labeling and localization, demonstrating the importance of temporal coherence in video data. In contrast, SETR [26] approaches semantic segmentation as a sequence-to-sequence task, using a pure transformer to encode images into patch sequences. This method provides global context and powerful segmentation capabilities without the need for convolutions, highlighting the versatility of transformer architectures. Furthering the efficiency in vision modeling, Swin Transformer [27] introduces a hierarchical architecture with shifted windows for efficient self-attention. This design enables scalable vision modeling with linear computational complexity relative to image size, addressing the computational challenges posed by large images. To address issues specific to large-vision models, Swin V2 [28] introduces residual-post-norm, log-spaced position bias, and SimMIM self-supervised pre-training. These innovations tackle training instability, resolution gaps, and the need for extensive labeled data. Swin-UNet [29] utilizes a pure Transformer architecture, combining hierarchical Swin Transformers based on shifted windows as the encoder and symmetric Swin Transformers as the patch-expanding layer decoder for high-performance image semantic segmentation. Swin-UNet overcomes the challenges of global–local feature learning and spatial resolution recovery and effectively alleviates the limitations of traditional Unet in processing large-scale images. SegNet [30] is known for its memory and computational efficiency, comprising encoder networks, decoder networks, and pixel-wise classification layers. Despite its low parameter cardinality and ease of training end-to-end, the max-pooling and subsampling may produce coarse segmentation results. Thus, SegNeXt [31] introduces the Multi-Scale Convolutional Attention (MSCA) structure, which leverages depth-wise separable convolutions, multi-branch depth-wise separable convolutions, and 1 × 1 convolutions in the decoder to construct a lightweight global context. This approach enhances semantic segmentation performance, addresses the scale diversity issues in scene understanding, and effectively mitigates the model’s reliance on information at different scales. DeepLabV3+ [32] is a deep, fully convolutional neural network that combines an encoder–decoder structure with an improved Atrous Spatial Pyramid Pooling (ASPP) module. DeepLabV3+ possesses strong semantic understanding capabilities, enabling it to better capture details through dilated convolutions and multi-scale contextual information. DeepLabV3-SAM [33] combines traditional semantic segmentation with the Segment Anything Model (SAM), integrating the zero-sample transfer capability with traditional algorithms to fully utilize SAM’s zero-sample transfer capability for low-cost, accurate, and fully automated image segmentation. LM-DeepLabv3+ [34] is a lightweight semantic segmentation method that adopts MobileNetV2 [35] as the backbone network and introduces the ECA-Net [36] attention mechanism and EPSA-Net [37] cross-dimensional channel attention to reduce the number of parameters and computational complexity while enhancing the feature representation capability and interaction of important features. In light of these advancements, a summary of the key neural network-based image segmentation methods and their contributions is presented in Table 2.

Despite the significant progress in semantic segmentation networks, these still suffer from insufficient contextual information, inadequate attention allocation, a lack of semantic information for low-level features, and insufficient resolution for high-level features. Therefore, this paper introduces an improved Deeplabv3+ model that utilizes ResNet101 as the backbone network. The proposed model incorporates a novel Semantic Channel Space Details Module (SCSDM) to extract richer information through multi-scale feature fusion and adaptive feature selection, enabling the network to focus on important features. Additionally, a Semantic Features Fusion Module (SFFM) is designed to integrate low-level and high-level semantic information better. Moreover, to address the problem of insufficient contextual information, the developed model utilizes a Dense Connection ASPP (DCASPP) module to enrich the feature representation through multiple convolutions, concatenations, and global average pooling operations. This strategy expands the feature sensory field and enhances semantic information.

1. A Dilated Convolutional Atrous Spatial Pyramid Pooling (DCASPP) module is constructed with multi-level dilated convolutions and global average pooling structures. In DCASPP, contextual information in semantic segmentation is extracted effectively, significantly enhancing the model’s understanding of complex scenes and segmentation accuracy.
2. SCSDM is introduced, which cleverly integrates the design of multi-scale receptive field fusion and spatial attention. SCSDM significantly enhances the model’s ability to perceive critical regions, thereby improving the accuracy and efficiency of semantic segmentation.
3. The SFFM is proposed, which incorporates feature-weighted fusion, channel attention, and spatial attention structures. This design effectively overcomes the challenges of limited semantic information in low-level features and reduced resolution in high-level features within semantic segmentation. Subsequently, the overall image comprehension and segmentation accuracy are significantly enhanced, demonstrating the SFFM’s crucial role in advancing the performance of semantic segmentation models.
4. Extensive experiments on two public datasets validate the effectiveness of SDAMNet. Specifically, SADMNet achieves a Mean Intersection over Union (MIOU) of 67.2% on the Aerial Semantic Segmentation Drone dataset and 75.3% on the UDD6 dataset.

The rest of this paper is organized as follows: Section 2 reviews the literature of the study. Section 3 illustrates the proposed SADMNet. Section 4 presents and discusses the experimental results. Finally, Section 5 provides the conclusions.

## 2. Related Work

This section introduces Deeplabv3++ and the attention mechanism, which are the major parts of the proposed SADMNet already available in the current literature.

### 2.1. Deeplabv3+

**Figure 1:**
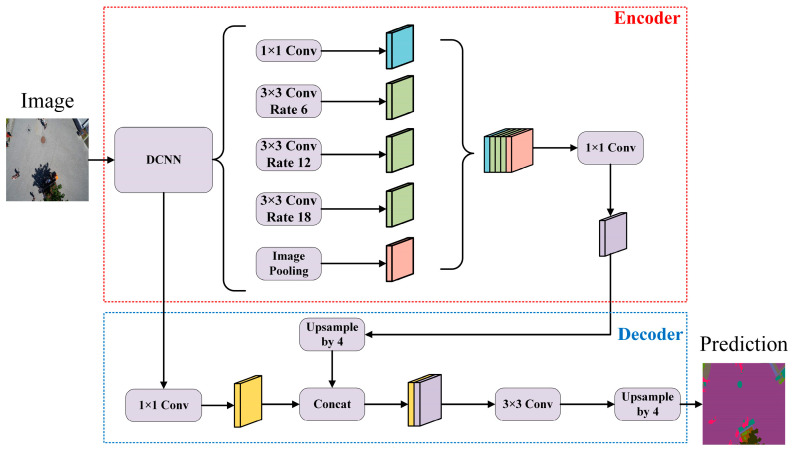
Overall DeepLabv3+ architecture.

Deeplabv3+ is a superior deep-learning model that integrates various innovative techniques to improve the accuracy and efficiency of image segmentation. Its architecture is illustrated in Figure 1, revealing that DeepLabv3+ adopts an encoder–decoder structure, fully leveraging the advantages of deep convolutional neural networks in image understanding. The encoder extracts high-level semantic features from the input image. At the same time, the decoder restores details and generates the final segmentation result by fusing multi-scale features and employing skip-connection techniques.

The encoder is a crucial component of the DeepLabv3+ network, comprising the backbone network ResNet101(DCNN) and the ASPP module. The encoder primarily aims to extract high-level semantic features from the input image, which are essential for understanding the semantic information and context of the image. ResNet101 [38], a deep convolutional neural network, is the backbone network and includes multiple convolution and pooling layers. These layers extract low-level features from the input images, such as edges and textures. In DeepLabv3+, ResNet101 functions as a feature extractor, processing the input image to form a feature map rich in high-level semantic information. The ASPP module is a vital component of DeepLabv3+, capturing multi-scale contextual information through parallel dilated convolutions, each with a different dilation rate. These branches introduce varying receptive fields in the convolution operation, capturing contextual information at various scales. The feature maps from all branches are then concatenated and fused along the channel dimension to create a rich multi-scale feature map. Finally, a 1 × 1 convolution reduces the number of feature channels, decreasing computational complexity.

The decoder is another essential component of the DeepLabv3+ network. It is responsible for restoring the feature maps from the encoder output to the original image size and performing feature fusion to generate the final semantic segmentation result. In the decoder, the underlying features obtained from the backbone network are first convolved with a 1 × 1 convolution to reduce the number of feature channels. This step decreases computation and memory usage for subsequent feature fusion operations. Then, the decoder performs a 4× upsampling on the multi-scale feature maps obtained from the encoder and concatenates them with the downsampled low-level features for fusion. This fusion process increases the number of channels in the feature maps by integrating feature information from different levels. After the feature fusion, the fused feature maps undergo a 4× upsampling through a 3 × 3 convolution to restore the maps to the size of the original input image. This process allows the network to generate segmentation results with high-level semantic information, labeling each pixel as a specific category and effectively accomplishing image semantic segmentation.

### 2.2. Attention Module

The attention mechanism is a widely used data processing method in machine learning that is used in various tasks, such as natural language processing, image recognition, and speech recognition. The attention mechanism mimics the observation mechanism of humans towards external entities. Generally, external information is attained by initially focusing on important local information and then integrating information from different regions when observing external entities. In other words, the attention mechanism allows machines to “pay attention” to the important parts as humans do, process, and understand data based on these important parts, thereby effectively performing various complex machine learning tasks. Spatial Attention [39] and SKNet [40] are two important attention mechanisms that will be introduced below.

**Figure 2:**
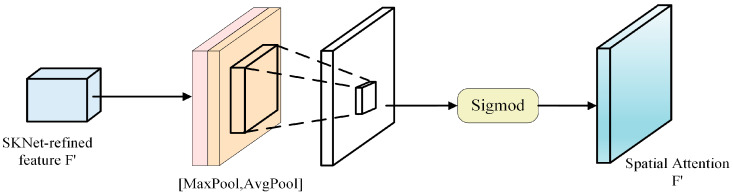
Spatial Attention module.

The Spatial Attention module utilizes a spatial attention mechanism, as depicted in Figure 2. Its core advantage lies in weighting features at different positions on the feature map, enabling the model to focus on important regions and enhance target localization accuracy. Spatial Attention introduces spatial attention weights in the channel dimension to improve its ability to perceive information from different spatial positions. For tasks such as semantic segmentation, Spatial Attention can focus on important spatial regions, thus improving segmentation accuracy and the overall model performance. However, it lacks operations on multi-scale feature maps and is insufficient in dealing with multi-scale feature representation and dynamic weighting.

**Figure 3:**
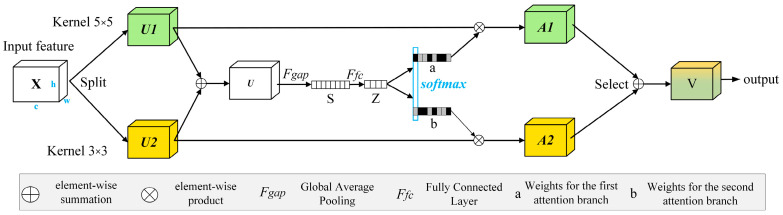
SKNet architecture.

Figure 3 presents SKNet, an upgraded version of the channel attention SENet. It optimizes task expressiveness by automatically learning and emphasizing features that are beneficial to the task while suppressing features that are irrelevant to the task. The module operates on input feature maps with convolutional kernels of different scales, obtaining multi-scale feature maps and extracting feature representations to varying scales through global information. Employing an adaptive approach to calculate the weight of each feature map achieves dynamic weighting of feature maps at various scales, thereby enhancing the model’s performance. S is the global pooled feature. Z is the reduced dimensional representation of S.

## 3. Semantic Segmentation Network Based on Adaptive Attention and Deep Fusion with the Multi-Scale Dilated Convolutional Pyramid

This section introduces the overall architecture of SDAMNet. Subsequently, the designed DCASPP, SCSDM, and SFFM are introduced.

### 3.1. Overall Architecture

**Figure 4:**
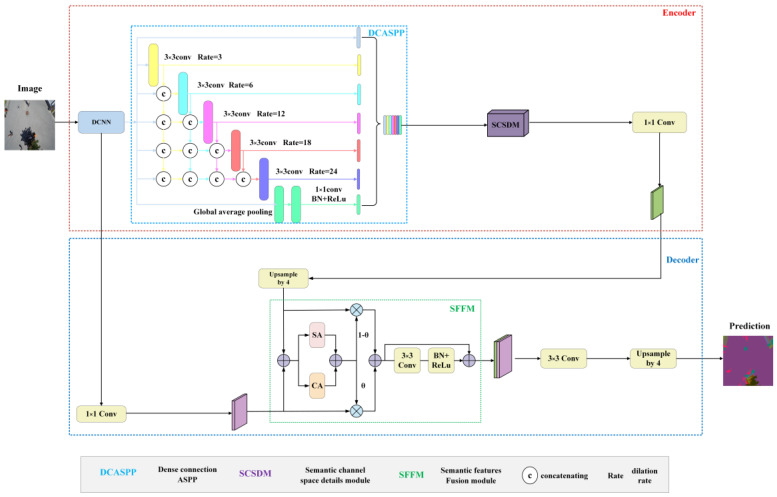
SDAMNet architecture.

**Figure 5:**
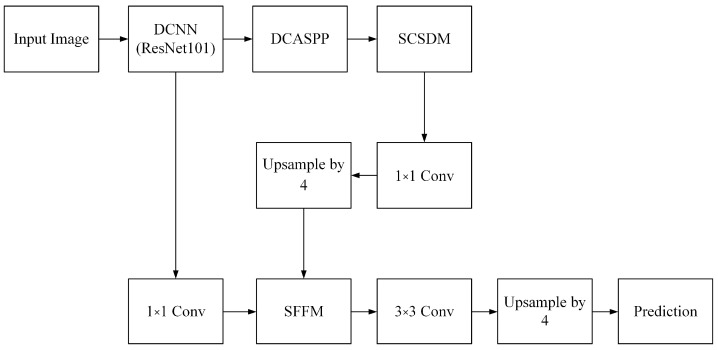
SDAMNet flowchart.

Although the segmentation network based on Deeplabv3+ has achieved good results, it suffers from two urgent problems: neglecting shallow features, leading to poor edge segmentation results, and decreasing the feature map resolution. The latter is due to multiple downsampling operations in deep convolutional neural networks, which reduce the prediction accuracy and discard boundary information. To tackle these issues, this paper develops a new semantic segmentation network named SDAMNet, with its architecture presented in Figure 4. The operation flowchart of SDAMNet is shown in Figure 5.

Figure 5 Simplified Summary of SDAMNet Algorithm

1. Encoder–Decoder Architecture
  SDAMNet is an end-to-end neural network utilizing an encoder–decoder architecture.
  Encoder: Uses ResNet101 as the backbone to extract multi-scale feature information.
  Decoder: Integrates high-level and low-level features, refines the feature maps using the SFFM module, adjusts channel numbers, processes with convolutions, upscales to the original image size, and enhances segmentation accuracy and detail capture.
2. Key Modules
  DCASPP (Dilated Convolutional Atrous Spatial Pyramid Pooling): Enhances feature extraction by capturing information at multiple scales and incorporating the global context.
  SCSDM (Semantic Channel Space Details Module): Improves the model’s ability to focus on important features and accurately capture target boundaries and details.
  SFFM (Semantic Features Fusion Module): Combines high-level and low-level features to utilize the strengths of both.
3. Processing Steps
  Feature Extraction: The encoder processes the input image to generate high-level and low-level semantic information.
  Feature Enhancement: The DCASPP and SCSDM modules refine the feature maps, enhancing the model’s perception of key areas and details.
  Feature Fusion: The SFFM module merges the enhanced feature maps, balancing the information from different levels.
  Upsampling and Prediction: The decoder adjusts the feature maps to the original input image size through upsampling, followed by the final prediction.

SDAMNet is an end-to-end neural network that adopts an encoder–decoder architecture. The encoder utilizes ResNet101 as the backbone, achieving multi-scale feature fusion through the DCASPP module and introducing global information aggregation. The SCSDM module further enhances the model’s ability to perceive important features and effectively captures target boundaries and details. The feature map is pre-processed by a 1 × 1 convolution to adjust the number of channels and an upsampling operation. The decoder achieves efficient feature fusion through the SFFM module, adaptively integrating the advantages of low-level and high-level features. The model’s semantic segmentation accuracy and effectiveness are substantially improved by comprehensively utilizing multi-scale features, introducing global information, and capturing detailed information.

Regarding the encoder of SDAMNet, the backbone ResNet101 generates two outputs, i.e., the high-level semantic information sent to the decoder and the low-level semantic information sent to the encoder. The output resolution of the high-level semantics is H/16 × W/16. H and W, respectively, represent the height and width of the input image. Moreover, the DCASPP module obtains rich feature information at various scales, and global average pooling integrates the global information of the entire image into the feature maps. Next, the obtained feature maps are input into the SCSDM module to further enhance the model’s perception of important features and its ability to focus on key areas. This architecture allows the segmentation results to capture the target boundaries and details more accurately, substantially enhancing the model’s semantic segmentation capability. Following this, channel cardinality in the feature map is reduced through 1 × 1 convolutions, which is then upsampled by ×4 and input into the decoder.

In the decoder, the output resolution of the underlying semantics is H/4 × W/4, and the number of channels of the underlying semantics is adjusted with a 1 × 1 convolution to match the number of channels in the feature map output by the encoder. Next, the SFFM module fuses the high-level and the low-level semantic feature maps, allowing it to adaptively and selectively fuse feature information from different levels, comprehensively utilizing the advantages from both low-level and high-level features. After that, the feature map is further processed through a 3 × 3 convolution operation, and the feature map is transformed to the original input image size by a ×4 upsampling process. Finally, the resized feature map is used for prediction. The whole process enables the model to capture detailed information better and improve the accuracy and effectiveness of semantic segmentation.

### 3.2. Dilated Convolutional Atrous Spatial Pyramid Pooling

The context information extraction module is crucial in semantic segmentation, significantly enhancing the model’s understanding of images and improving segmentation accuracy. Introducing a larger receptive field and enhancing the contextual correlation capabilities enhances the neural network’s understanding of the overall image structure and the relationship between pixels. The context information extraction module is particularly effective for handling complex scenes, size variations, and pixel details. Having an understanding of the global context enables the model to perceive distant objects and related areas, while multi-scale feature fusion helps to address different scales and semantic dependencies. At the same time, it reduces spatial information loss, enhances semantic features, and significantly improves the segmentation effect.

This paper introduces the DCASPP context information extraction module to enhance the model’s understanding of the overall image structure and pixel relationships and address the demands of complex scenes and multi-scale features. The DCASPP module in SDAMNet in Figure 4 shows the structure in detail, where “c” denotes concatenation and “Rate” is the dilation rate. This module further strengthens the acquisition and utilization of context information, enhancing the model’s capability in complex scenes and processing image details.

The output of DCASPP is mathematically formulated as follows. Let *x* be the input feature map. The concatenation operation is defined as Concat(a,b) concatenating feature maps a and b along the channel dimension. The dilated convolution operation *Conv_d_*(*x*) refers to the convolution operation with a dilation rate of d, the global average pooling operation is *GAP*(*x*), and *ReLU*(*Norm*(*Conv*1×1(*x*))) involves a 1 × 1 convolution, normalization, and a ReLU activation function. Finally, *Upsample*(*x*, *size*) is the bilinear interpolation upsampling operation.

(1) y1=Concat(x,Convd=3(x))y2=Concat(y1,Convd=6(y1))y3=Concat(y2,Convd=12(y2))y4=Concat(y3,Convd=18(y3))g=ReLU(Norm(Conv1×1(GAP(x))))g″=Upsample(g,size=x)y=Concat(x,Convd=3(x),Convd=6(y1),Convd=12(y2),Convd=18(y3),Convd=24(y4),g″)

Initially, the input feature map *x* undergoes a convolution operation with a dilation rate of 3, resulting in a new feature map. This new feature map is concatenated with the original input feature map *x*, forming a richer feature representation. The concatenated feature map is then passed to the next layer module as input, where a convolution operation with a dilation rate of 6 is performed. The result of this convolution is concatenated with the previously concatenated feature map to continue integrating multi-scale semantic information. Following this, the concatenated result from the previous step is used as input for convolution operations with dilation rates of 12, 18, and 24 successively, with each result concatenated with the previously concatenated result, continuing this integration process.

The input feature map *x* subsequently undergoes global average pooling to obtain a feature map containing the overall contextual information. The feature map generated by global average pooling is processed by applying a 1 × 1 convolution, followed by normalization and ReLU activation to adjust the feature values. To integrate the global information with the previous results, bilinear interpolation is employed to upsample the result of global average pooling to the same size as the input feature map *x*. This upsampled global pooling feature map is concatenated with the previous concatenation result.

Ultimately, a feature map is obtained that integrates multi-scale dilation rate information and global contextual information. This feature map exhibits a more robust expressive capability in semantic segmentation, enabling better comprehension of semantic information for objects at different scales in the image and providing more accurate prediction results for segmentation.

Multiple convolutions, concatenations, and global average pooling processes gradually enrich the feature representation throughout the process, expanding the feature-receiving field and enhancing the semantic information.

### 3.3. Semantic Channel Space Details Module

**Figure 6:**
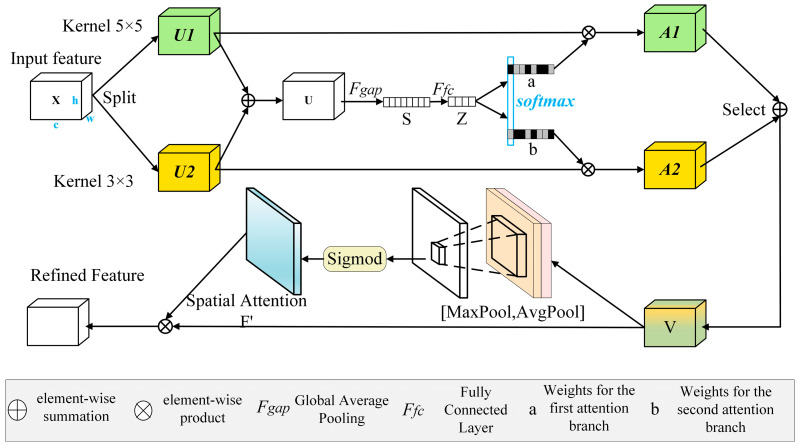
SCSDM module.

Attention mechanisms are essential for high-quality semantic segmentation. They force the model to focus on important regions relevant to the task, reduce background interference, and enhance segmentation accuracy. However, they lack operations on multi-scale feature maps and thus cannot handle feature representations and dynamic weighting across different scales. Therefore, the SCSDM attention module (Figure 6) is proposed to address these two limitations simultaneously.

The original feature map X is convolved with kernels of different sizes, producing the corresponding feature maps *U*1 and *U*2, representing information from different receptive fields. Then, *U*1 and *U*2 are summed to obtain the fused feature map U, integrating information from multiple receptive fields. U has a size of [*c*, *h*, *w*], where c denotes the number of channels and h and w denote the height and width. Next, global average pooling is applied to the feature map *U* along the height and width dimensions to obtain channel-level information, forming a one-dimensional tensor of size [*c*, 1, 1], indicating the importance of each channel.

(2) $$\begin{array}{l} U=U1+U2\\ s_{c}=\frac{1}{H\times W}\sum_{i=1}^{H}\sum_{j=1}^{W}U_{C}(i,j) \end{array}$$

where *U* is the fused feature map, *U*1 and U2 are the feature maps obtained by convolving with kernels of different sizes, and *s_c_* is the global average pooling value of channel *c*.

A linear transformation maps the original C-dimensional information to the D-dimensional space, aiming to process this information further. Subsequently, another linear transformation remaps the D-dimensional information back to the original C-dimensional space, completing the information extraction in the channel dimension:

(3) z=δ(B(Ws))

where *z* is the transformed compact feature vector, *δ* is the ReLU activation function, *B* is the batch normalization operation, *W* is the weight matrix, and *s* is the channel-level information tensor. Next, the Softmax function normalizes the attention scores, providing a set of attention scores, with each channel corresponding to a score indicating its importance, i.e., *a_c_* and *b_c_*. The resulting attention scores are multiplied with the corresponding *U*1 and *U*2 to obtain *V*:

(4) $$V=a_{c}\cdot U1+b_{c}\cdot U2,a_{c}+b_{c}=1$$

where *V* is the generated feature map.

Applying attention weighting to the feature maps at various scales provides the final feature map *V*, which integrates information from different receptive fields. Compared to the initial feature map *X*, module *V* has refined information integrated from multiple receptive fields, enhancing the model’s ability to perceive key regions in semantic segmentation and thereby improving the accuracy and performance of the segmentation process.

Next, *V* undergoes average pooling to obtain *avg_out* and simultaneously undergoes max pooling to obtain *max_out*. These operations are defined as follows:

(5) $$\begin{array}{l} avg\_out=AvgPool(V)\\ max\_out=MaxPool(V) \end{array}$$

The resulting *avg_out* and *max_out* are concatenated to form *V*′, which undergoes a convolution operation to learn the attention weights for different spatial positions:

(6) $$V^{\prime \prime }=Conv(V^{\prime })$$

The output *V*″ is then normalized using the sigmoid function to obtain the attention weights attention_weights for different spatial positions:

(7) $$attention\_weights=\sigma (V^{\prime \prime })$$

where *σ* represents the sigmoid function.

By applying these attention weights to the input feature map *V*, each channel is multiplied by the corresponding attention weight to introduce spatial attention:

(8) $$V_{final}=V\times attention\_weights$$

This spatial attention mechanism enhances the model’s perception capability and prioritizes information from different spatial positions within the input feature map *V*. Consequently, during tasks such as semantic segmentation, the model focuses more effectively on crucial spatial regions, improving segmentation accuracy and effectiveness.

Ultimately, through the aforementioned operations, the multi-scale receptive field information and attention weights from different spatial positions are integrated, enhancing the model’s perception of crucial regions. This further improves semantic understanding and segmentation capabilities, improving overall performance.

### 3.4. Semantic Features Fusion Module

Feature fusion for semantic segmentation is crucial, as it effectively integrates features from different levels or branches and addresses issues such as the lack of semantic information in low-level features and the lower resolution of high-level features. Feature fusion enables the model to comprehensively capture image details and semantic information, significantly improving segmentation performance, particularly in handling complex scenes and details. Feature fusion is also crucial in deep learning due to improving performance in object detection and image segmentation.

**Figure 7:**
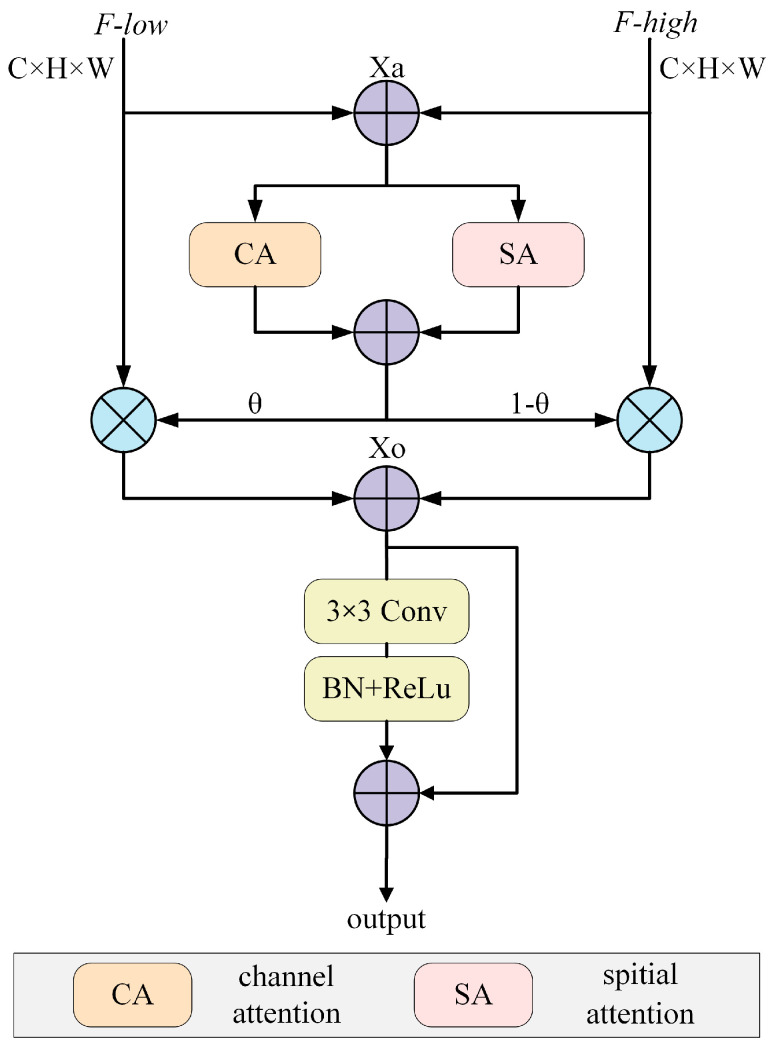
SFFM architecture.

However, to further optimize the feature fusion process, this paper develops the SFFM fusion module illustrated in Figure 7. SFFM correlates features at different levels and combines the information of different scales and semantics to improve the quality of feature fusion and enhance the model’s performance in the semantic segmentation task. This design philosophy fully leverages the advantages of the features, effectively compensating for their deficiencies and enhancing the model’s ability to capture target shapes and semantic information, thus improving the performance of semantic segmentation models.

The output of SFFM is mathematically expressed as follows:

(9) $$\begin{array}{l} xa=F\text{-}\mathrm{low}⊕F\text{-}\mathrm{high}\\ c=CA(xa)\\ s=SA(xa)\\ wei=c⊕s\\ xo=x⊗wei⊕y⊗(1-wei)\\ xi=xo⊕conv\_layers(xo) \end{array}$$

The following content provides a detailed explanation of the above formula. The SFFM first performs an addition operation on the input features to obtain the fused feature xa. Then, the Channel Attention (CA) module computes the channel attention weights and aggregates feature information through pooling and convolutional operations to enhance the perception of crucial semantic information. Additionally, CA aids in accurately distinguishing pixels of different categories, thereby enhancing the segmentation accuracy. Moreover, the Spatial Attention (SA) module computes the spatial attention weights. Specifically, by applying average pooling and max pooling, spatial information is captured. Convolutional operations then generate attention maps emphasizing the importance of various spatial locations, thereby enhancing the perception of features at different scales. This is important, as enhancing the model’s perception of object shapes and positions improves the accuracy and robustness of semantic segmentation models.

Furthermore, merging channel attention and spatial attention provides the attention map θ. Then, by multiplying the low-level semantic features with the attention map, multiplying the high-level semantic features with 1-θ, and adding them together, the weighted features xo are obtained. The weighted features xo contain the information fused from the channel and spatial attention and are passed through a convolutional layer for convolutional operation, further extracting features and enhancing the model’s representational capacity. Adding them aims to integrate the feature information emphasized by the attention mechanism with the feature information extracted by the convolutional operation, thus obtaining a richer and more accurate semantic representation.

The entire design concept and structure enable the model to integrate low-level and high-level features effectively. Furthermore, by focusing on information from different channels and spatial positions through the channel and spatial attention, the model enhances its performance in semantic segmentation. This process improves the perception of features at different scales and positions, thereby enhancing accuracy and generalization capability.

## 4. Experiments

This section first describes the dataset used in the experiments. Next, it provides implementation and evaluation details. Subsequently, ablation studies are conducted to validate and demonstrate the modules’ effectiveness. Finally, the proposed model is compared with state-of-the-art models to confirm its advantages.

### 4.1. Datasets

#### 4.1.1. Aerial Drone Image Dataset

**Figure 8:**
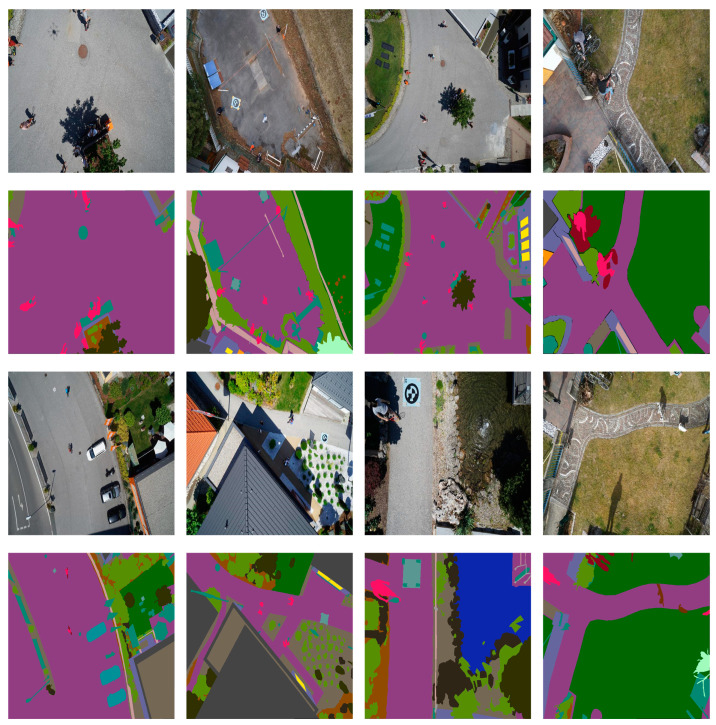
Sample images of the Aerial Drone Image dataset.

The Aerial Drone Image dataset [41] is an open-source aerial drone image dataset available on Kaggle, which includes 24 different categories. This dataset aims to improve the semantic understanding and safety of autonomous UAV flight and landing sequences in urban scenarios. The images in this dataset are captured from an aerial perspective, depicting areas ranging from 5 to 30 m above the ground and containing over 20 buildings. Each image is captured by a high-resolution camera with a resolution of 6000 × 4000 pixels. Focusing on urban scenes, only a small number of individual images from limited categories are retained, resulting in 400 images. For training and evaluating the machine learning models, the dataset is divided into training, validation, and testing sets with a ratio of 8:1:1. Figure 8 depicts some sample images, where the first and third rows are the original images, and the second and fourth rows are the corresponding label images.

#### 4.1.2. UDD6 Dataset

**Figure 9:**
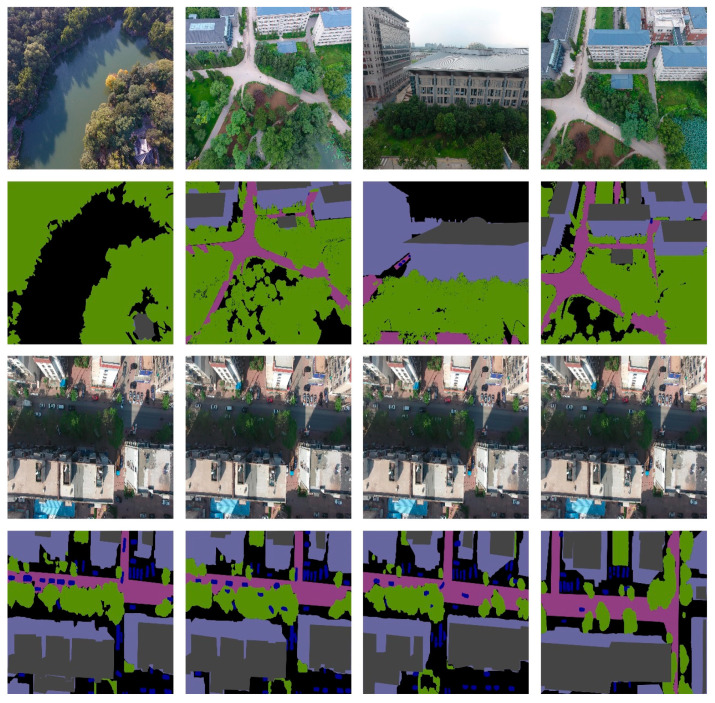
Sample images of the UDD6 dataset.

The UDD6 dataset [42] contains image samples captured by drones within the range of 60 to 100 m, with a resolution of 4096 × 2160 or 4000 × 3000 pixels. The UDD6 dataset comprises 160 image samples with segmentation labels, capturing six classes of objects in urban scenes, such as facades, roads, vegetation, vehicles, and roofs. The dataset is divided into training, validation, and test sets with a ratio of 8:1:1 to train and evaluate the machine learning models. Figure 9 displays some sample images, where the first and third rows represent the original images, and the second and fourth rows depict their corresponding label images.

### 4.2. Implementation Details and Evaluations

#### 4.2.1. Experimental Setup

For objectivity, the proposed model is implemented in PyTorch to employ the same data augmentation techniques. The model is trained on an NVIDIA A40 GPU with 48 GB of VRAM, sourced from NVIDIA Corporation, headquartered in Santa Clara, California, United States. Moreover, the Stochastic Gradient Descent (SGD) [43] optimizer is utilized for model evaluation to expedite processing speed and minimize the loss function. The initial learning rate is set to 0.014. Table 3 reports the other hyperparameters.

#### 4.2.2. Evaluation Indicators

Mean Intersection over Union (MIOU) is a standard metric in semantic segmentation and is crucial for recent segmentation tasks. In semantic segmentation, Intersection over Union (IOU) measures the ratio of the intersection over the union between the true labels and the predicted values for each class. Frequency-Weighted IOU (FWIOU) is a slight improvement over MIoU. Unlike MIoU, FWIOU assigns weights based on the frequency of occurrence of each class, making it an important evaluation metric in practical semantic segmentation problems. Accuracy (ACC) indicates the overall correctness of the model’s pixel classification. It represents the proportion of correctly classified pixels (including true positives and true negatives) out of the total number of pixels. Using ACC as a metric directly measures the model’s effectiveness in accurately segmenting different classes in an image, thereby aiding in the evaluation of the model’s performance. The higher the ACC value, the better the model’s overall accuracy in recognizing and annotating various objects and scenes in aerial images. The performance of all the models is evaluated on the datasets presented in Section 4.1.1 and Section 4.1.2, based on the accuracy, MIOU, and FWIOU metrics, which are defined as follows:

(10) $$\begin{array}{l} \mathrm{MIOU}=\frac{1}{k+1}\sum_{i=0}^{k}\frac{\mathrm{TP}}{\mathrm{TP}+\mathrm{FP}+\mathrm{FN}}\\ \mathrm{ACC}=\frac{\mathrm{TP}+\mathrm{TN}}{\mathrm{TP}+\mathrm{FP}+\mathrm{TN}+\mathrm{FN}}\\ \mathrm{FWIOU}=\frac{\mathrm{TP}+\mathrm{FN}}{\mathrm{TP}+\mathrm{FP}+\mathrm{TN}+\mathrm{FN}}\frac{\mathrm{TP}}{\mathrm{TP}+\mathrm{FP}+\mathrm{FN}} \end{array}$$

where TP, TN, FP, and FN are the true positives, true negatives, false positives, and false negatives, respectively.

### 4.3. Experimental Results

Extensive experiments are conducted on two image semantic segmentation datasets to explore the effectiveness of the designed model.

#### 4.3.1. Ablation Study on DCASPP

This section delves into DCASPP’s influence on the network’s performance and its importance in model improvement. For this case, the Deeplabv3+ model is selected as the baseline model, and the ASPP structure in Deeplabv3+ is replaced with DCASPP. The effectiveness of DCASPP is validated through several experiments on two different datasets. Table 4 reports the experimental results, providing an intuitive understanding of DCASPP’s contribution to network performance.

**Figure 10:**
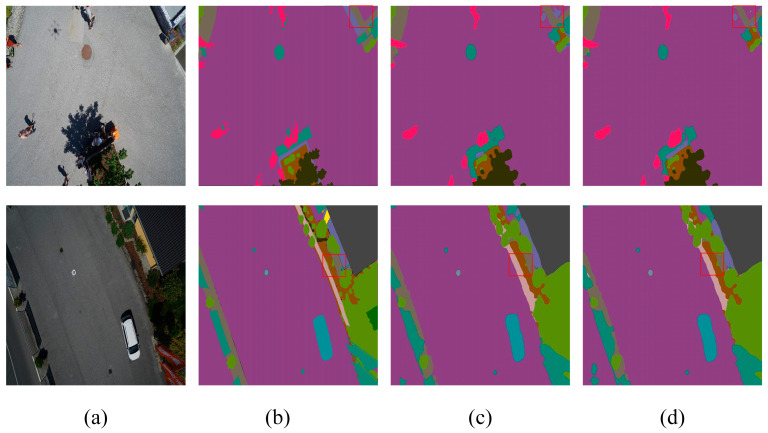
Aerial Drone Image dataset—ablation study on DCASPP (**a**) original image, (**b**) mask, (**c**) Deeplabv3+, and (**d**) Deeplabv3+-DCASPP. The red squares highlight the comparative effectiveness of the module in specific regions.

**Figure 11:**
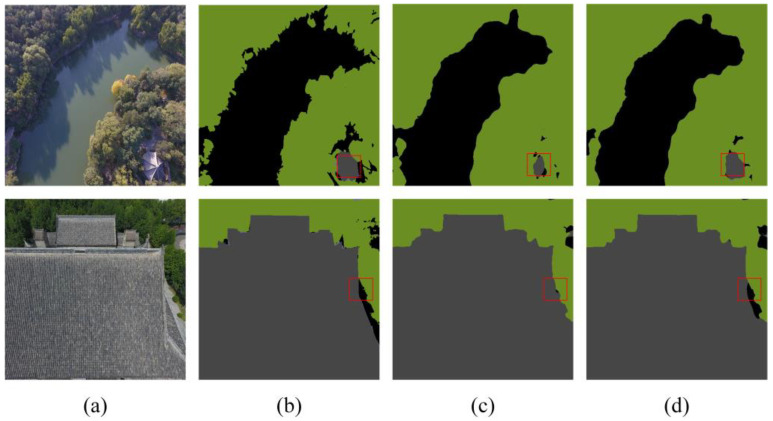
UDD6 images dataset—ablation study on DCASPP (**a**) original image, (**b**) mask, (**c**) Deeplabv3+, and (**d**) Deeplabv3+-DCASPP. The red squares highlight the comparative effectiveness of the module in specific regions.

Table 4 highlights that DCASPP achieves the highest ACC, MIOU, and FWIOU on the Aerial Drone Image dataset, improving performance by 0.22%, 0.71%, and 0.32%, respectively, compared to the baseline network (Deeplabv3+). On the UDD6 dataset, DCASPP demonstrates the best performance across all evaluation metrics. These results indicate that DCASPP has significant advantages in understanding complex scenes and multi-scale features, thereby enabling more accurate predictions in semantic segmentation. Sample images for both datasets are provided to validate the experimental results, as depicted in Figure 10 and Figure 11. The contents of the red boxes in these figures reveal that using the Deeplabv3+-DCASPP method can effectively segment and remove some edges that should not have existed in the first place.

This visual evidence supports the quantitative improvements reported in Table 4. The above experiments, along with the visualization results, fully validate the effectiveness of the DCASPP method. By integrating multi-scale convolution and global contextual information extraction, DCASPP effectively combines features and enhances semantic expression. This process corrects segmentation errors and results in more detailed and accurate segmentation outcomes. Figure 10 and Figure 11 demonstrate the superior performance of DCASPP in handling complex scenes, which is particularly evident in the improved boundary delineation and feature extraction.

#### 4.3.2. Ablation Study on SCSDM

To assess the effectiveness of SCSDM, the segmentation results of Deeplabv3+ are compared with those of Deeplabv3+ enhanced with SCSDM. The corresponding outcomes are presented in Table 5.

Table 5 reveals that on the Aerial Drone Image dataset, the MIOU and FWIOU values of Deeplabv3+ are 64.28% and 89.09%, while Deeplabv3+SCSDM attains 65.49% and 89.69%, respectively, and improves ACC by 0.4%. Furthermore, on the UDD6 dataset with SCSDM, the ACC, MIOU, and FWIOU increased by 0.8%, 0.79%, and 0.43%, respectively.

**Figure 12:**
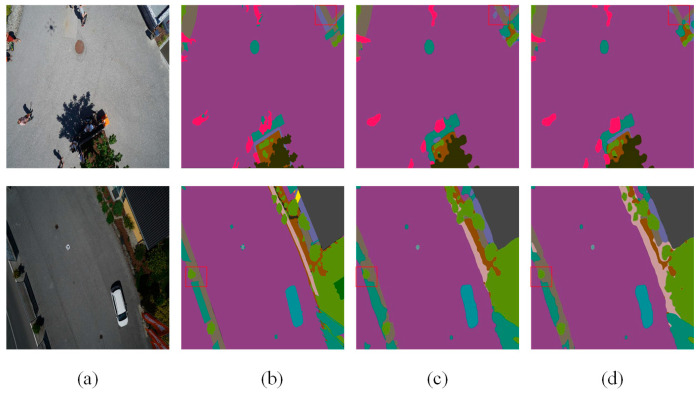
Aerial Drone Image—ablation study on SCSDM (**a**) original image, (**b**) mask, (**c**) Deeplabv3+, and (**d**) Deeplabv3+-SCSDM. The red squares highlight the comparative effectiveness of the module in specific regions.

**Figure 13:**
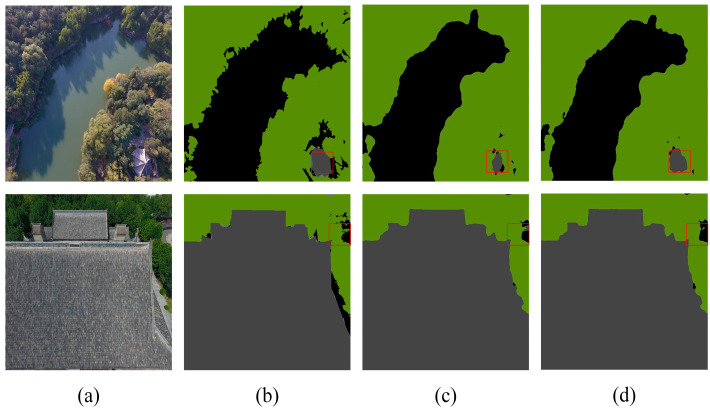
UDD6 dataset—ablation study on SCSDM (**a**) original image, (**b**) mask, (**c**) Deeplabv3+, and (**d**) Deeplabv3+-SCSDM. The red squares highlight the comparative effectiveness of the module in specific regions.

These quantitative improvements are visually corroborated in Figure 12. The graphical representations demonstrate that SCSDM enhances the segmentation detail along edges, effectively segmenting and eliminating initially unnecessary edges. Moreover, Figure 13 shows that SCSDM achieves more accurate segmentation than Deeplabv3+ alone. This can be attributed to the unique advantages of SCSDM, which integrates multi-scale receptive field information and attention weights at different spatial positions, thereby significantly enhancing the model’s ability to perceive critical regions.

In summary, the enhancements to all scalar metrics of the network further evidence the effectiveness and utility of SCSDM. The advantage exhibited by SCSDM lies in handling edge details and identifying and correcting anomalous edges. Through this forward-looking mechanism, the model demonstrates superior performance in complex scenes, resulting in breakthroughs in image segmentation. Therefore, these experimental results fully validate the potential of SCSDM as an effective segmentation model component, providing strong support for research and applications in image segmentation.

#### 4.3.3. Ablation Study on SFFM

This section discusses the impact and significance of SFFM on the network performance. Deeplabv3+ is chosen as the baseline model, and the feature fusion structure in Deeplabv3+ is replaced with SFFM. The effectiveness of SFFM is evaluated on the same two datasets utilized in the previous trials. Table 6 presents the corresponding results demonstrating the impact of SFFM on network performance and highlighting its importance in the model architecture.

**Figure 14:**
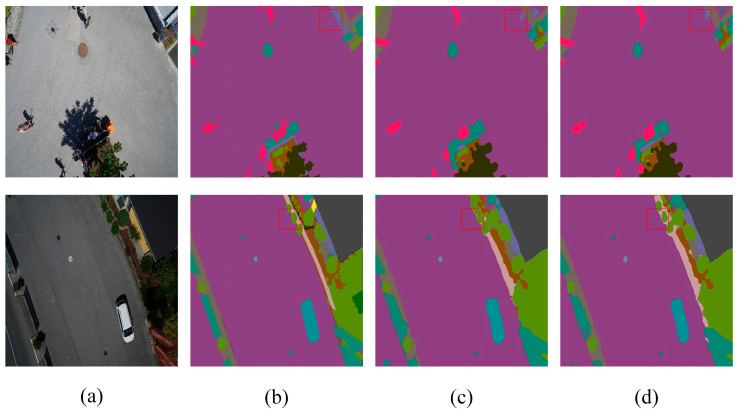
Aerial Drone Image dataset—ablation study on SFFM (**a**) original image, (**b**) mask, (**c**) Deeplabv3+, and (**d**) Deeplabv3+-SFFM. The red squares highlight the comparative effectiveness of the module in specific regions.

**Figure 15:**
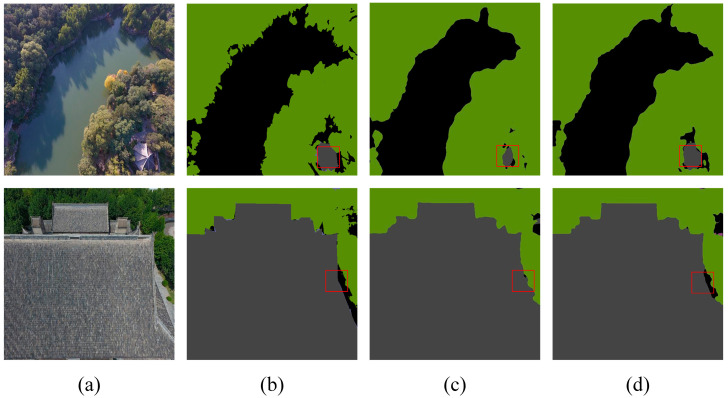
UDD6 dataset—ablation study on SFFM (**a**) original image, (**b**) mask, (**c**) Deeplabv3+ image, and (**d**) Deeplabv3+-SFFM Image. The red squares highlight the comparative effectiveness of the module in specific regions.

The results in Table 6 indicate that in the Aerial Drone Image dataset, the SFFM achieved improvements of 0.23%, 0.92%, and 0.39% in ACC, MIOU, and FWIOU, respectively, compared to the Deeplabv3+ fusion module. This demonstrates that the SFFM enhances feature extraction performance to a certain extent. Figure 14 and Figure 15 depict the experimental results for two sample images, illustrating this ablation study’s outcomes. In these images, the regions indicated by red boxes show that the SFFM achieves better segmentation results. This is attributed to its capability to effectively integrate low-level and high-level features and utilize channel and spatial attention to focus on information from different channels and spatial positions.

In the UDD6 dataset, the SFFM improves the model’s performance by 0.83%, 0.80%, and 0.67% in ACC, MIOU, and FWIOU, respectively, compared to the Deeplabv3+ methods, confirming the effectiveness of the SFFM. Overall, the experimental results of the Deeplabv3+-SFFM method on the two datasets reveal that introducing the SFFM leads to more precise edge information segmentation, thus improving the model’s performance. This improvement also demonstrates good generalization value on the Aerial Drone Image dataset.

Additionally, the visual results in Figure 14 and Figure 15 further support the quantitative findings, showcasing the SFFM’s superior ability to achieve finer segmentation details compared to the baseline model.

Furthermore, the effectiveness of the SFFM feature fusion module is evaluated on the Aerial Drone Image dataset. Specifically, different fusion methods are compared, including the concatenation channel concatenation method, channel addition method, and the proposed SFFM method. Table 7 presents the experimental results to provide a more intuitive understanding of SFFM’s performance in network performance. The results highlight that on the Aerial Drone Image dataset, SFFM has a significantly improved performance compared to that of the concatenation and addition fusion methods. This suggests that utilizing SFFM can improve feature extraction performance.

The data in Table 7 show that the SFFM method achieves the highest performance across all three metrics. Specifically, the Mean Intersection over Union (MIOU) is improved by approximately 0.92% compared to the concatenation method and 1% compared to the addition method. Similarly, the Accuracy (ACC) sees an increase of 0.23% and 0.23%, respectively, while the Frequency Weighted Intersection over Union (FWIOU) improves by 0.39% and 0.41%. These improvements, although seemingly incremental, are significant in the context of image segmentation tasks where even minor enhancements can lead to better feature extraction and overall model performance. This suggests that utilizing SFFM can substantially improve feature extraction performance, leading to more accurate and reliable results in aerial drone image analysis.

#### 4.3.4. Ablation Study on the Overall SDAMNet Architecture

The effectiveness of the proposed model is demonstrated through comprehensive experiments on the two datasets used during the ablation studies. Table 8 reports the experimental results on the Aerial Drone Image dataset, and Table 9 reports the corresponding results on the UDD6 dataset.

Table 8 reveals that as the model is progressively built with its components, its performance improves on all metrics. Finally, by incorporating all models into Deeplabv3+, the MIOU, ACC, and FWIOU reach 67.17%, 94.65%, and 90.45%, respectively, demonstrating the optimal semantic segmentation capability compared to the baseline Deeplabv3+. Hence, introducing the DCASPP, SFFM, and SCSDM modules significantly improves all metrics, i.e., 2.89% in MIOU, 0.83% in ACC, and 1.36% in FWIOU compared to Deeplabv3+.

**Figure 16:**
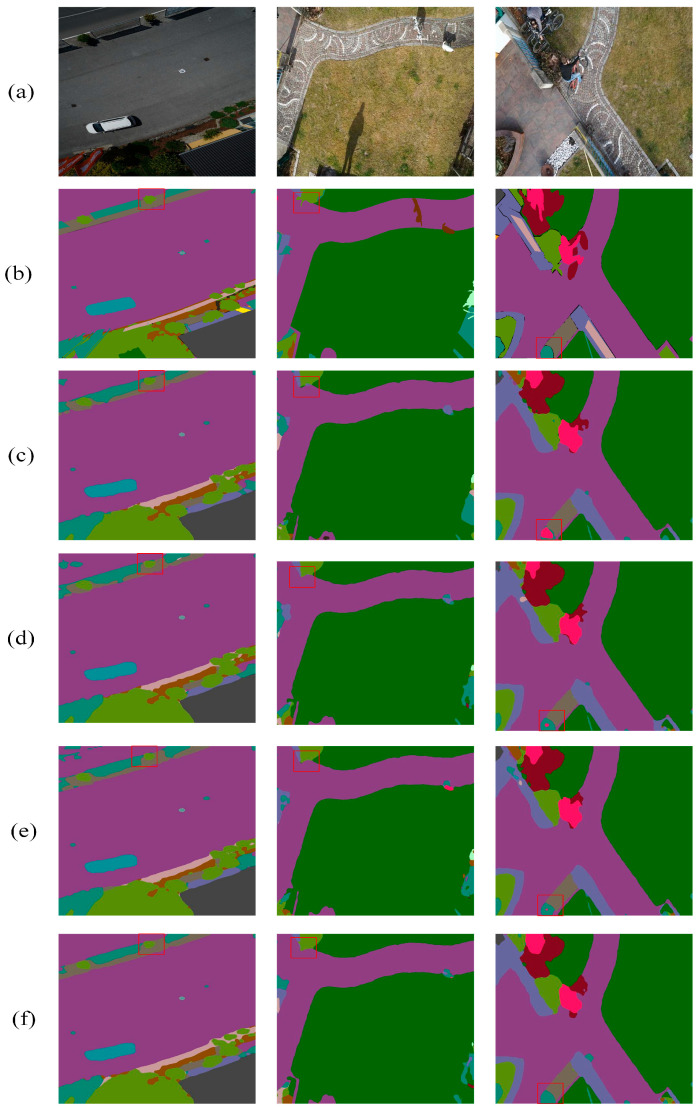
Results on the Aerial Drone Image dataset (**a**) original image, (**b**) mask, (**c**) Deeplabv3+, (**d**) Deeplabv3+ with DCASPP, (**e**) Deeplabv3+ with DCASPP and SFFM, and (**f**) SDAMNet. The red squares highlight the comparative effectiveness of the module in specific regions.

**Figure 17:**
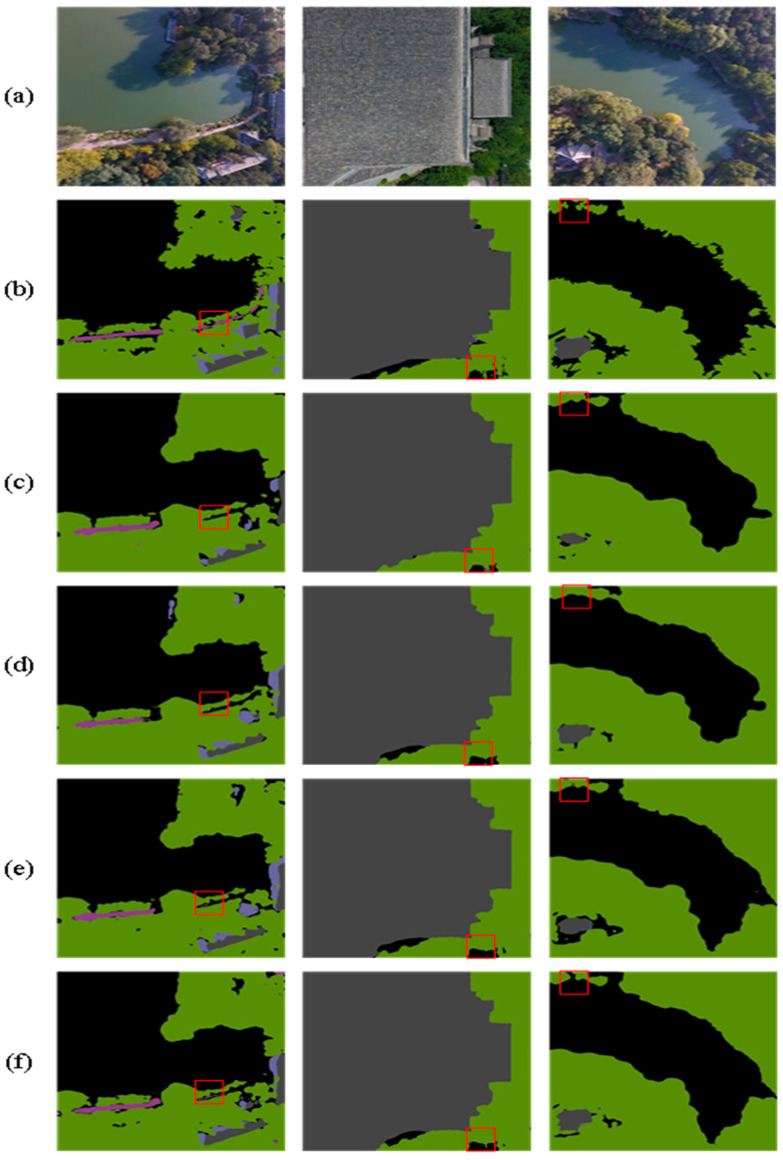
Results on the UDD6 dataset (**a**) original image, (**b**) mask, (**c**) Deeplabv3+, (**d**) Deeplabv3+ with DCASPP, (**e**) Deeplabv3+ with DCASPP and SFFM, and (**f**) SDAMNet. The red squares highlight the comparative effectiveness of the module in specific regions.

Regarding the UDD6 dataset, by incorporating all modules into Deeplabv3+, the performance enhancement becomes 2.13% in MIOU, 2.11% in ACC, and 1.57% in FWIOU. Figure 16 and Figure 17 illustrate the results more clearly in two example images, which reveal a progressive improvement in processing details from top to bottom. The enhancement primarily stems from the three introduced modules that enable the model to accurately understand the overall structure of the image, improve segmentation accuracy, and enhance the perception of critical regions.

Additionally, they better capture the shape and semantic information of targets, thereby comprehensively improving the performance of semantic segmentation models. These results fully demonstrate that combining Deeplabv3+ with DCASPP, SFFM, and SCSDM strengthens the model’s semantic segmentation capability on both datasets.

#### 4.3.5. Evaluation of the Aerial Drone Image Dataset

This section compares SDAMNet with several state-of-the-art methods, including DANet [15], SETR [26], Swin [27], SwinV2 [28], Twins [19], BiMSANet [18], UVid-Net [25], and HMANet [20] on the Aerial Drone Image dataset. The segmentation data are presented in Table 10.

The results in Table 8 reveal that SDAMNet achieves an MIOU of 67.2% in semantic segmentation while DANet, SETR, Swin, SwinV2, Twins, BiMSANet, UVid-Net, and HMANet attain an MIOU of 63.8%, 58.6%, 63.1%, 64.2%, 65.3%, 65.5%, 65.8%, and 63.5%, respectively. Thus, in semantic segmentation, SDAMNet demonstrates higher accuracy than some advanced methods, with its advantage ranging from 1.4% to 8.6% in terms of segmentation accuracy.

**Figure 18:**
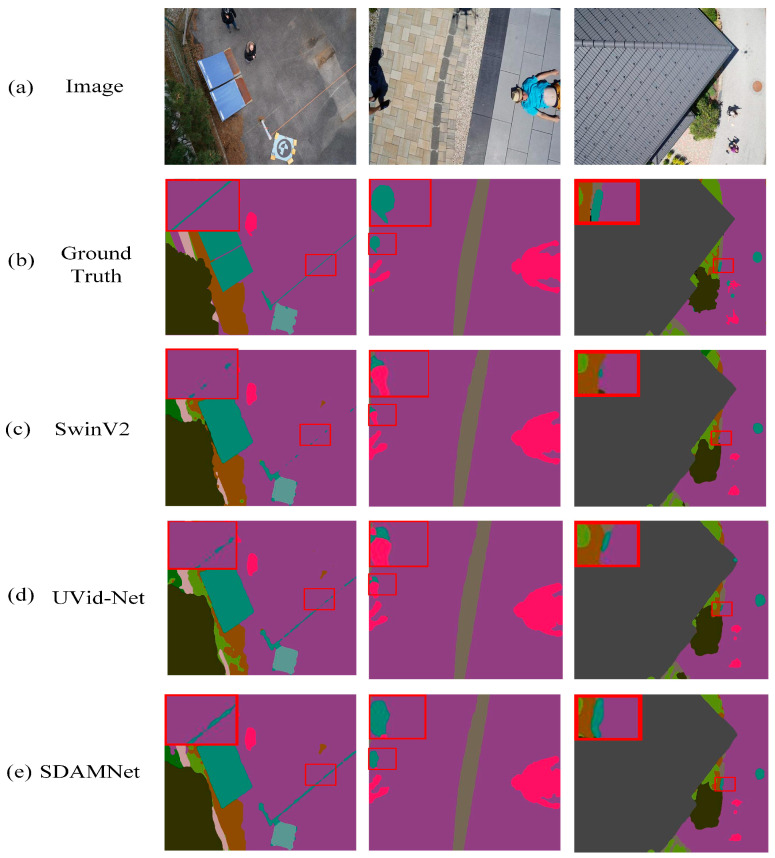
Results on the UDD6 images (**a**) original RGB color image, (**b**) ground truth label, (**c**) SwinV2, (**d**) UVid-Net, and (**e**) SDAMNet. The red squares highlight the comparative effectiveness of the model in specific regions.

Figure 17 illustrates comparative segmentation results on the Aerial Drone Image dataset. The red rectangular boxes highlight some areas with better segmentation results. Figure 18 infers that the edges segmented by SDAMNet are more refined, and SDAMNet successfully segments and removes edges that should not exist. The notable enhancement can be primarily credited to SDAMNet’s characteristics, which integrate multi-scale receptive field information and spatial positional attention. This combination enhances the model’s perception of key areas. Besides, by integrating multi-scale and global contextual information, the model’s understanding of the overall image structure and pixel correlations is improved.

#### 4.3.6. Comparison of the UDD6 Dataset

SDAMNet is also compared with several state-of-the-art methods on the UDD6 dataset, including DANet [15], ACNet [16], OCRNet [17], SETR [26], Swin [27], and Segformer [24]. The segmentation data are provided in Table 11.

The results in Table 11 reveal that SDAMNet achieves an MIOU of 75.3% in semantic segmentation while the competing state-of-the-art methods, such as DANet, ACNet, OCRNet, SETR, Swinformer, and Segformer achieve MIOU values of 73.7%, 74.1%, 73.9%, 71.9%, 72.7%, and 74.9%, respectively. Hence, SDAMNet exhibits higher segmentation accuracy than some advanced methods, with its advantage ranging from 0.4% to 3.4% in terms of MIOU.

**Figure 19:**
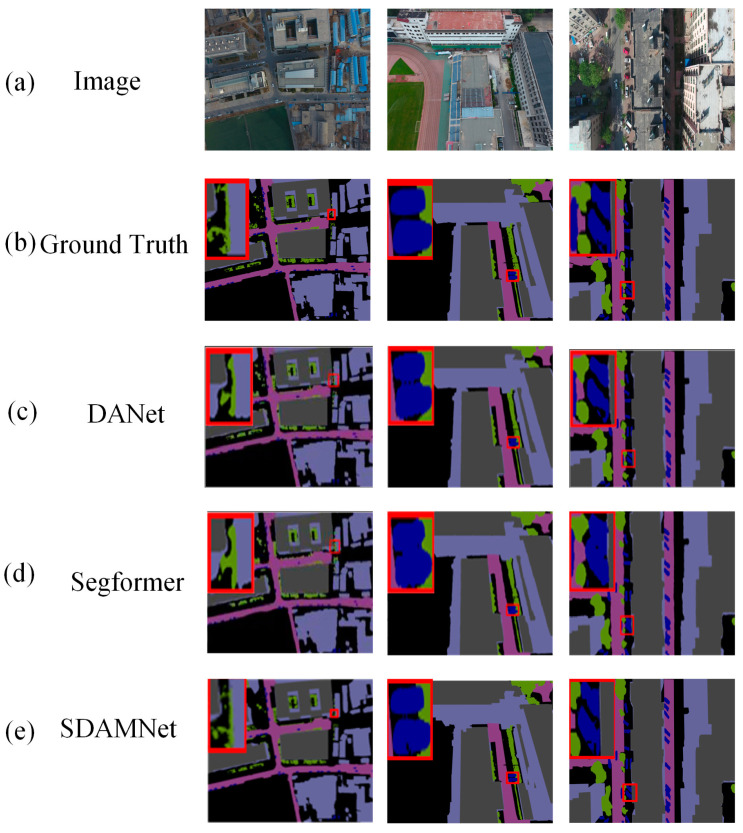
Visualization results of different methods on the UDD6 dataset (**a**) original RGB color image, (**b**) ground truth labels, (**c**) DANet, (**d**) Segformer, and (**e**) SDAMNet. The red squares highlight the comparative effectiveness of the model in specific regions.

Figure 19 visualizes the results of the proposed model, along with two other methods, DANet and Segformer, using the UDD6 dataset. On the UDD6 dataset, six classes of objects are primarily annotated with different colors for color labeling. Additionally, some areas with better segmentation results are highlighted with red dashed rectangles. Figure 19 clearly illustrates that only SDAMNet achieves finer segmentation and thus excels in recognizing object shapes and positional information, demonstrating that the spatial detail branch preserves the object’s positional information. Considering the comprehensive comparison of the UDD6 dataset, SDAMNet achieves higher segmentation accuracy by effectively integrating the key modules introduced in this paper.

The average processing time (inference time) per UAV image is an important consideration for urban scene segmentation. To compare the real-time performance of various networks, quantitative experiments are conducted, and the model parameters and floating-point operations (FLOPs) are evaluated to determine the practicality of the networks. Table 12 presents a comparison of computational efficiency, model weights, and computational complexity.

According to the data analysis in Table 12, Deeplabv3+ exhibits the best performance in real-time applications with the fastest inference time (55.0 ms) and the smallest model parameters (63 MB), although its accuracy is relatively low. The inference times of DANet and ACNet are 55.6 ms and 56.4 ms, with model parameters of 69 MB and 72 MB, respectively. While suitable for real-time applications, their computational complexity is relatively high, with 1836 G and 1938 G FLOPs. OCRNet has an inference time of 58.6 ms, model parameters of 83 MB, and 2036 G FLOPs. Segformer’s inference time is 60.4 ms, with 168 MB of model parameters and 2158 G FLOPs. SDAMNet achieves a good balance between real-time performance and accuracy with an inference time of 59.3 ms, model parameters of 91 MB, and 2075 G FLOPs. Swin has a higher inference time (65.2 ms), with model parameters of 234 MB and 2336 G FLOPs, suitable for scenarios with abundant computational resources. SETR has the longest inference time (77.8 ms), with the largest model parameters and computational complexity at 308 MB and 3018 G FLOPs, respectively. Although it may have higher accuracy, it is unsuitable for real-time applications. Overall, SDAMNet provides a good balance, suitable for scenarios requiring a combination of real-time performance and accuracy.

## 5. Conclusions

To achieve better accuracy and performance in semantic segmentation, a semantic segmentation network called SDAMNet was designed based on the structure of Deeplabv3+. Specifically, a DCASPP module was designed to address the challenges of complex scenes, scale variations, and details in semantic segmentation by leveraging multi-scale feature fusion and global contextual understanding. Moreover, to tackle the challenges of focusing on task-critical regions and balancing local details with global semantic information in semantic segmentation, an SCSDM module was devised. This module innovatively integrates attention weights of multi-scale receptive field information and spatial positions, effectively enhancing the accuracy and efficiency of semantic segmentation. Furthermore, to capture image details and semantic information more comprehensively, the SFFM was designed. Channel and spatial attention mechanisms are utilized by this module to skillfully fuse features from different levels and channels. As a result, segmentation performance is improved, the perception of target shapes and positions is enhanced, and segmentation accuracy and generalization capability are increased. Extensive experiments and qualitative analysis validate the effectiveness of the SDAMNet method, showing improvements in MIOU by 2.89% and 2.13% on the tested datasets. However, this study has some limitations. The current model’s computational efficiency and inference time need optimization to ensure its practical applicability in real-world scenarios. Additionally, the method’s performance across diverse datasets and its adaptability to different semantic segmentation tasks require further exploration. Future work will focus on refining the methodology, including experimenting with different attention mechanisms and feature extraction through multiple paths to explore more task-appropriate designs. This will aim to enhance model efficiency and applicability in various semantic segmentation tasks. Researchers can also explore applying SDAMNet to image segmentation tasks in other domains, improving attention mechanisms, and integrating multimodal data to further enhance its practical value. Furthermore, optimizing the network’s computational efficiency, reducing model inference time, and utilizing automated machine learning techniques to automatically find the optimal network architecture will be key areas for future research.

## Figures and Tables

**Table 1:** Summary of traditional image segmentation methods.

Method	Year	Key Contributions
Threshold-Based	1975	Classifies pixels into foreground and background by setting a grayscale threshold. Simple but poor on complex images.
Edge-Based	2011	Detects differences in grayscale values between pixels and forms edge contours. Sensitive to noise.
Region-Based	2009	Segments images based on spatial information and pixel similarity features. Sensitive to noise.

**Table 2:** Summary of neural network-based image segmentation methods.

Method	Year	Key Contributions
AlexNet	2012	Employs multiple convolutions to extract local features, significantly improving segmentation beyond traditional methods.
BiSeNet	2018	Introduces a detail branch for extracting details and boundary information, and a semantic branch for capturing global contextual information, enriching spatial features.
BiSeNetV2	2021	Enhances BiSeNet with a more powerful detail branch and a lightweight spatial branch, achieving better performance and real-time inference speed.
DANet	2019	Integrates local and global features using spatial and channel attention modules, enhancing feature representation for precise segmentation.
ACNet	2019	Utilizes Asymmetric Convolution Blocks (ACBs) to replace standard convolutions, improving accuracy and robustness against distortions.
OCRNet	2021	Aggregates object-contextual representations to enhance pixel classification by leveraging the relationship between pixels and object regions.
BiMSANet	2021	Tackles scale variation in oblique aerial images using bidirectional multi-scale attention networks for more effective semantic segmentation.
Twins	2021	Revisits spatial attention design, proposing Twins-PCPVT and Twins-SVT for optimized matrix multiplications.
HMANet	2021	Integrates class-augmented and region shuffle attention to enhance VHR aerial image segmentation.
DDRNet	2021	Fuses features through bidirectional and dense connections, introducing a multi-scale feature fusion technique.
U-Net	2015	Known for its encoder–decoder architecture, using skip connections to fuse low-level and deep-level information.
U2-Net	2020	Adopts a two-level nested U-shaped structure with Residual U blocks (RSU) to capture more contextual information, reducing training costs.
SegFormer	2021	Unifies Transformers with MLP decoders, using a hierarchically structured encoder and lightweight decoder for efficient multi-scale feature aggregation.
UVid-Net	2021	Enhances UAV video semantic segmentation by incorporating temporal information and a feature-refiner module for accurate, temporally consistent labeling.
SETR	2021	Uses a pure transformer to encode images into patch sequences, providing global context and powerful segmentation capabilities.
Swin Transformer	2021	Introduces a hierarchical architecture with shifted windows for efficient self-attention, addressing computational challenges of large images.
Swin V2	2022	Introduces residual-post-norm, log-spaced position bias, and SimMIM self-supervised pre-training to tackle training instability and resolution gaps.
Swin-UNet	2022	Combines hierarchical Swin Transformers as the encoder and symmetric Swin Transformers as the decoder for high-performance image semantic segmentation.
SegNet	2017	Known for memory and computational efficiency, but max-pooling and subsampling may produce coarse segmentation results.
SegNeXt	2022	Introduces Multi-Scale Convolutional Attention (MSCA) structure with depth-wise separable convolutions for enhanced semantic segmentation performance.
DeepLabV3+	2018	Combines an encoder–decoder structure with an improved Atrous Spatial Pyramid Pooling (ASPP) module for strong semantic understanding capabilities.
DeepLabV3-SAM	2023	Integrates the Segment Anything Model (SAM) with traditional semantic segmentation algorithms for accurate, low-cost, and fully automated image segmentation.
LM-DeepLabv3+	2024	Lightweight method using MobileNetV2 as the backbone network, with ECA-Net and EPSA-Net attention mechanisms to reduce parameters and computational complexity while enhancing feature representation.

**Table 3:** Hyperparameter setup.

Value	Aerial Drone Image Dataset	UDD6 Dataset
Batch size	8	4
Optimizer	SGD	SGD
Learning strategy	CosineAnnealingLR	CosineAnnealingLR
Epochs	150	150
Batch size	8	4

**Table 4:** Ablation study of DCASPP.

	MIOU (%)	ACC (%)	FWIOU (%)
Methods on the Aerial Drone Image Dataset			
Deeplabv3+	64.28	93.82	89.09
Deeplabv3+DCASPP	64.99	94.04	89.41
Methods on the UDD6 Dataset			
Deeplabv3+	73.14	86.91	79.26
Deeplabv3+DCASPP	73.67	87.41	79.47

**Table 5:** Ablation study on SCSDM.

	MIOU (%)	ACC (%)	FWIOU (%)
Methods on the Aerial Drone Image Dataset			
Deeplabv3+	64.28	93.82	89.09
Deeplabv3+SCSDM	65.49	94.22	89.69
Methods on the UDD6 Dataset			
Deeplabv3+	73.14	86.91	79.26
Deeplabv3+SCSDM	73.93	87.71	79.69

**Table 6:** Ablation study of SFFM.

	MIOU (%)	ACC (%)	FWIOU (%)
Methods on the Aerial Drone Image Dataset			
Deeplabv3+	64.28	93.82	89.09
Deeplabv3+-SFFM	65.20	94.05	89.48
Methods on the UDD6 Dataset			
Deeplabv3+	73.14	86.91	79.26
Deeplabv3+-SFFM	73.94	87.74	79.93

**Table 7:** Feature fusion comparison experiments.

	MIOU (%)	ACC (%)	FWIOU (%)
concat	64.28	93.82	89.09
add	64.20	93.82	89.07
SFFM	65.20	94.05	89.48

**Table 8:** Ablation experiments on the Aerial Drone Image dataset.

Deeplabv3+	DCASPP	SFFM	SCSDM	MIOU (%)	ACC (%)	FWIOU (%)
√				64.28	93.82	89.09
√	√			64.99	94.04	89.41
√	√	√		65.48	94.19	89.68
√	√	√	√	67.17	94.65	90.45

**Table 9:** Ablation experiments on the UDD6 dataset.

Deeplabv3+	DCASPP	SFFM	SCSDM	MIOU (%)	ACC (%)	FWIOU (%)
√				73.14	86.91	79.26
√	√			73.67	87.41	79.47
√	√	√		74.19	87.81	80.08
√	√	√	√	75.27	89.02	80.83

**Table 10:** Comparison of the Aerial Drone Image dataset.

Method	MIOU (%)
Deeplabv3+	64.3
DANet	63.8
SETR	58.6
Swin	63.1
SwinV2	64.2
Twins	65.3
BiMSANet	65.5
UVid-Net	65.8
HMANet	63.5
SDAMNet	67.2

**Table 11:** Comparison of the UDD6 dataset.

Method	MIOU (%)	mAcc (%)
Deeplabv3+	73.1	86.9
DANet	73.7	87.5
ACNet	74.1	87.8
OCRNet	73.9	87.7
SETR	71.9	85.5
Swin	72.7	86.3
Segformer	74.9	88.1
SDAMNet	75.3	89.0

**Table 12:** Comparison of computational efficiency, model weights, and computational complexity.

Method	Inference Time (ms)	Model Parameter (MB)	FLOPs (G)
Deeplabv3+	55.0	63	1232
DANet	55.6	69	1836
ACNet	56.4	72	1938
OCRNet	58.6	83	2036
SETR	77.8	308	3018
Swin	65.2	234	2336
Segformer	60.4	168	2158
SDAMNet	59.3	91	2075

## Data Availability

The data presented in this study can be requested from the corresponding author, and these data are not currently available for public access.

## Nomenclature

Abbreviation	Full Term
SA	Spatial Attention
CA	Channel Attention
Rate	Dilation Rate
ASPP	Atrous Spatial Pyramid Pooling
SDAMNet	Semantic Segmentation Network Based on Adaptive Attention and Deep Fusion with the Multi-Scale Dilated Convolutional Pyramid
DCASPP	Dilated Convolutional Atrous Spatial Pyramid Pooling
SCSDM	Semantic Channel Space Details Module
SFFM	Semantic Features Fusion Module
MIOU	Mean Intersection over Union
FWIOU	Frequency Weighted Intersection over Union
ACC	Accuracy
ReLU	Rectified Linear Unit
BN	Batch Normalization
SGD	Stochastic Gradient Descent
TP	True Positive
FP	False Positive
TN	True Negative
FN	False Negative
